# Serotonin-secreting enteroendocrine cells respond via diverse mechanisms to acute and chronic changes in glucose availability

**DOI:** 10.1186/s12986-015-0051-0

**Published:** 2015-12-15

**Authors:** Leah Zelkas, Ravi Raghupathi, Amanda L. Lumsden, Alyce M. Martin, Emily Sun, Nick J. Spencer, Richard L. Young, Damien J. Keating

**Affiliations:** Department of Human Physiology and Centre for Neuroscience, Flinders University, Sturt Rd, Adelaide, SA 5042 Australia; South Australian Health and Medical Research Institute (SAHMRI), Adelaide, 5001 Australia; Discipline of Medicine, University of Adelaide, Adelaide, SA 5001 Australia

**Keywords:** Enteroendocrine, Glucose, Serotonin

## Abstract

**Background:**

Enteroendocrine cells collectively constitute our largest endocrine tissue, with serotonin (5-HT) secreting enterochromaffin (EC) cells being the largest component (~50 %). This gut-derived 5-HT has multiple paracrine and endocrine roles. EC cells are thought to act as nutrient sensors and luminal glucose is the major absorbed form of carbohydrate in the gut and activates secretion in an array of cell types. It is unknown whether EC cells release 5-HT in response to glucose in primary EC cells. Furthermore, fasting augments 5-HT synthesis and release into the circulation. However, which nutrients cause fasting-induced synthesis of EC cell 5-HT is unknown. Here we examine the effects of acute and chronic changes in glucose availability on 5-HT release from intact tissue and single EC cells.

**Methods:**

We utilised established approaches in our laboratories measuring 5-HT release in intact mouse colon with amperometry. We then examined single EC cells function using our published protocol in guinea-pig colon. Single cell Ca^2+^ imaging and amperometry were used with these cells. Real-time PCR was used along with amperometry, on primary EC cells cultured for 24 h in 5 or 25 mM glucose.

**Results:**

We demonstrate that acute increases in glucose, at levels found in the gut lumen rather than in plasma, trigger 5-HT release from intact colon, and cause Ca^2+^ entry and 5-HT release in primary EC cells. Single cell amperometry demonstrates that high glucose increases the amount of 5-HT released from individual vesicles as they undergo exocytosis. Finally, 24 h incubation of EC cells in low glucose causes an increase in the transcription of the 5-HT synthesising enzyme Tph1 as well as increasing in 5-HT secretion in EC cells.

**Conclusions:**

We demonstrate that primary EC cells respond to acute changes in glucose availability through increases in intracellular Ca^2+^ the activation of 5-HT secretion, but respond to chronic changes in glucose levels through the transcriptional regulation of Tph1 to alter 5-HT synthesis.

## Background

Enteroendocrine cells collectively constitute our largest endocrine tissue, made up of cells that are dispersed throughout the GI tract epithelium. There are multiple different enteroendocrine cell types, each synthesizing different hormones, with some containing multiple hormones [1, 2]. Almost all our body’s 5-HT is synthesized in enteroendocrine cells called enterochromaffin (EC) cells [3]. 5-HT plays a crucial role in enteric neurotransmission, the propagation of intrinsic enteric reflexes, multiple gastrointestinal disorders and a range of homeostatic mechanisms [4, 5]. EC cells supply all our circulating 5-HT and recent evidence has demonstrated that plasma 5-HT is essential for metabolic homeostasis [6] and obesity [7, 8]. However, despite the important physiological roles of 5-HT, the mechanisms by which EC cell 5-HT release is controlled remains poorly understood.

Due to their location, enteroendocrine cells are exposed to a unique environment, including ingested nutrients. Luminal glucose plays an important role in the GI tract, where concentrations up to 200–300 mM are estimated to exist across the luminal brush border membrane after a meal [9]. Glucose is the major form of absorbed carbohydrate and acts as a signal for the activation of many regulatory events. The presence of glucose in the intestinal lumen stimulates a number of changes in GI function including inhibition of both gastric emptying [10] and food intake [11] and stimulation of pancreatic secretion [12]. There is also evidence that activation of extrinsic vagal afferent neurons by intestinal glucose can be mediated by release of endogenous 5-HT and activation of 5-HT_3_ receptors located on vagal afferent nerve terminals [10, 13], which subsequently slows gastric emptying and reduces food intake [11].

It is currently unknown whether glucose itself acts directly on primary EC cells or activates 5-HT release via secondary mechanisms involving other cell types, such as through gut contraction, in the intact tissue preparations used [14, 15]. BON cells, a cell line derived from a metastatic human carcinoid tumour of the pancreas, are responsive to relatively high levels of glucose (50–75 mM) [16]. However, as these are not primary EC cells and are derived from the pancreas, where no EC cells exist, it is not known how closely BON cell function represents EC cell function. Moreover evidence that 2-deoxyglucose triggers 5-HT release from human ileal EC cells {Kidd, 2008 #2007} should be interpreted with caution, as this glucose analogue can inhibit cell glycolysis. Accordingly, it is essential to investigate whether luminal glucose has a direct effect on EC cell 5-HT release.

Nutrients not only trigger 5-HT release post-prandially, they may also have more chronic effects on 5-HT synthesis. Fasting was recently demonstrated to augment plasma 5-HT levels through a mechanism involving the increased expression of Tph1, the rate-limiting enzyme in EC cell 5-HT synthesis [6]. However which nutrient changes are involved in this fasting-induced increase in 5-HT synthesis remains unknown. In this study we approach the key questions of whether acute and chronic changes in glucose availability affect EC cell 5-HT release using primary EC cells and intact tissue. We find that glucose levels of 100 mM are required to trigger EC cell 5-HT release, that this causes Ca^2+^ entry in primary EC cells, and that the mechanism of action involves a significant increase in the amount of 5-HT released from individual vesicles. We also observe that chronic changes in glucose availability (24 h) alter 5-HT release via a different mechanism involving the transcription of Tph1 and 5-HT synthesis and release.

## Methods

### Primary culture and purification of EC cells

Guinea pigs (350–500 g) were euthanized humanely by stunning with a blow to the head followed by severing of the carotid arteries, as approved by the Animal Welfare Committee at Flinders University. EC cell isolation was undertaken as previously described [17]. 4–6 cm of distal colon tissue was removed and a midline incision made along the lining of the colon and then pinned mucosal-side uppermost in a dish containing Krebs solution (in mM: 140 NaCl, 5 KCl, 2 CaCl_2_, 1 MgCl_2_, 10 Hepes, 5 D-glucose, pH 7.4. The mucosal layer was scraped off, minced and centrifuged at 1000 × *g* for 5 min. The tissue was digested with 0.05 % Trypsin-EDTA (Sigma Aldrich USA) containing 1 mg/ml Collagenase A (Roche Diagnostics Germany) at 37 °C for 30 min with continuous agitation. The reaction was then stopped by adding an equal volume of growth medium (DMEM containing 10 % FBS, 1 % L-glutamine and 1 % penicillin–streptomycin, Sigma Aldrich USA) The suspension was filtered through a 40 μm filter and centrifuged at 1000 × *g* for 5 min and the resulting pellet was resuspended in 1 ml of growth medium. Density gradient centrifugation was then performed to separate different cell populations in order to purify EC cells. The density gradient was created by layering Percoll solutions of varying densities with the dense end at the bottom of the tube (according to manufacturer’s instructions). The cell fraction was then layered on top of the Percoll density gradient. Centrifugation was performed at 1100 × *g* for 8 min with slow braking. EC cells were harvested at a density of 1.07 g/ml, and then washed once with growth medium and plated into 6 cm^2^ pre-treated cell culture dishes (Iwaki, Australia). EC cells were maintained in growth medium for 2–4 days at 37 °C.

### Immunocytochemistry

EC cells were grown for 24 h on glass coverslips pre-treated with 10 μg/ml of poly-d-lysine (Sigma Aldrich USA) in growth medium. Cells were fixed for 18–20 h in Zamboni’s fixative at 4 °C followed by serial 5 min washes: 4 × 80 % EtOH, 2 × 100 % EtOH, 3 × DMSO, 4 × PBS. Fixed cells were incubated for 30 min in 10 % normal donkey serum diluted in antibody diluent (290 mM NaCl, 7.5 mM Na_2_HPO_4_, 2.6 mM Na_2_HPO_4_.2H2O, 0.1 % NaN_3_ in distilled water, pH 7.1) at room temperature. Cells were then incubated with a goat monoclonal antibody against 5-HT (Jackson Immunoresearch USA, 1:750 in antibody diluent) for 24 h at room temperature in a humid chamber. After 3 × 5 min washes with PBS, the cells were incubated with donkey anti-goat IgG tagged to the fluorescent label Cy3 (Jackson Immunoresearch USA, 1:200 in antibody diluent) and the nuclear marker DAPI (Sigma Aldrich USA, 1:500) for 2 h at room temperature in a humid chamber. After 3 × 5 min washes with PBS, the coverslips were mounted onto glass slides in buffered glycerol. Fluorescence was visualised on an Olympus BX50 Fluorescence Imaging Microscope under 40x magnification. Fluorescence was shown as an overlay of Cy3 (green) and DAPI (blue) fluorescence.

### Calcium imaging

Ca^2+^ imaging was undertaken as previously reported [18]. EC cells were treated with Fluo-4 AM (4 μM) in Krebs solution for 30 min at 37 °C. Cells were maintained in Krebs solution and stimulated with Krebs solution containing either, 100 mM K^+^, 100 mM Glucose, 20 mM Glucose or 10 μM Acetylcholine. Calcium influx was visualised as increases in cell fluorescence on a Cascade II fluorescent microscope (Photometrix, USA). Results were analysed on Imaging Workbench software (version 6.0.22) (INDEC BioSystems, Inc, USA).

### Amperometric measurement of 5-HT release in single cells

5-HT release from single EC cells was measured using amperometry [19–21]. A carbon-fibre electrode (5-μm diameter, ProCFE, Dagan Corporation, USA) was placed one micron above a single EC cell. 400 mV was applied to the electrode and the current caused by the oxidation of 5-HT was recorded using an EPC-9 amplifier and Pulse software (HEKA Electronic, Germany). Krebs solution was the standard bath solution and the temperature was controlled using an automatic temperature controller at 34 °C–36 °C (TC-344B; Warner Instrument Corporation, Hamden, CT). All solutions were applied to cells using a gravity perfusion system. Single EC cells were perfused with Krebs solution for 30 s then stimulated for 60 s with Krebs containing 70 mM KCl (replacing an equimolar amount of NaCl).

### Measurement of 5-HT release in intact tissue

The distal colon was removed from C57/Bl6 mice and placed in Krebs solution oxygenated with 95 % O_2_, 5 % CO_2_. A midline incision was made along the lining of the colon and then was pinned mucosal-side uppermost in Sylgard-lined organ bath containing oxygenated Krebs solution. The colon was placed mucosa uppermost in Sylgard-lined gel in an organ bath and was continuously perfused with oxygenated Krebs solution. The temperature was controlled using an automatic temperature controller at 34 °C–36 °C (TC-344B; Warner Instrument Corporation, Hamden, CT). Serotonin release was measured in intact tissue using amperometry as previously described [22, 23]. A carbon-fibre electrode (5-μm diameter, ProCFE; Dagan Corporation, Minneapolis, MN), was lowered a few microns above the epithelial layer to avoid contact with the tissue surface or mucosa during contractions, therefore 5-HT release would not be stimulated by the electrode compressing the tissue. 400 mV was applied to the electrode and the current caused by the oxidation of 5-HT was recorded using an EPC-7 amplifier and Pulse software (HEKA Electronic, Lambrecht/Pfalz, Germany), sampled at 10 kHz and low-pass filtered at 1 kHz. Secretion events were determined as events in which the amplitude increased more than 10 times the root-mean-squared noise of the baseline. Amplitude of each event was taken as the current size of the peak relative to baseline. Recordings and analysis were undertaken in a paired fashion to minimise potential variance between different tissue preparations or over time. Thus, we analysed the effect under control (10 mM glucose) and then experimental (30, 50 or 100 mM glucose) and compared this statistically to their own control data run, prior to each high glucose exposure.

### Measurement of Tph1 expression in guinea pig EC cells

Guinea pig EC cells were purified as described above and grown in plates for 24 h in growth medium containing either 25 mM or 5 mM glucose. Cells were harvested and washed once with DMEM containing no glucose. Total RNA was extracted using the RNeasy Mini kit (Qiagen Australia) according to manufacturer’s instructions. RNA quality was analysed on a Nanodrop1000 spectrophotometer and samples were only used if the A_260_/A_280_ ratio was greater than1.9. cDNA was prepared from the RNA samples using the Omniscript RT kit (Qiagen Australia) and random hexamers (Life Technologies Australia) according to manufacturer’s instructions. qPCR was carried out using a Quantitect SYBR Green kit (Qiagen Australia) on a Rotorgene 3000 according to manufacturer’s instructions as previously described [24]. Samples were run in triplicate and data from at least 3 different cell cultures was analysed using the REST-RG spreadsheet [25]. Results were normalized to 18S rRNA as a housekeeping gene. Primers for the qPCR were designed using using the PrimerQuest Online Primer Design tool (Integrated DNA Technologies, http://www.idtdna.com/Scitools/Applications/Primerquest/) and independently confirmed the results using Primer-BLAST (http://www.ncbi.nlm.nih.gov/tools/primer-blast/index.cgi). Primers used were (5’-3’): TPH1 (F-TTGGTTTGTGCAAGCAGGAC, R-AGTGGAGGTTGGAGTTCACTG) AND 18S rRNA (F-TGCATGGCCGTTCTTAGTTG, R-AGTTAGCATGCCAGAGTCTCGTT).

### Amperometry measurements and statistics

Amperometry files were converted to Axon Binary Files (ABF Utility, version 6.0.1, Synaptosoft, USA) and secretory spikes were analysed (Mini Analysis, version 6.0.1, Synaptosoft, USA) from before and during stimulation [26, 27]. Amperometric spikes were selected for analysis of event frequency if spike amplitude exceeded 10 times root-mean-squared noise of the baseline. Rise time of each spike was calculated from the 50–90 % rising phase. The number of each individual spike per second before and during stimulation was measured in Hz. Data in the results section are present as mean ± S.E.M. The number of molecules of 5-HT (N) released per exocytotic event from single cells was calculated using the formula N = Q/nF where Q is the charge in coulombs, n is the number of electrons donated by 5-HT upon oxidation and F is the Faraday constant in Coulombs [28].

### Statistical analysis

Differences were considered statistically significant when *P* <0.05. A paired *t*-test was used to compare individual data sets from the same sample. Unpaired t–tests were used to analyse data from single cell recordings. A Mann–Whitney test was used to compare non-parametric spike analysis data.

## Results and discussion

### High glucose causes 5-HT release in intact colonic tissue preparations

**Fig. 1:**
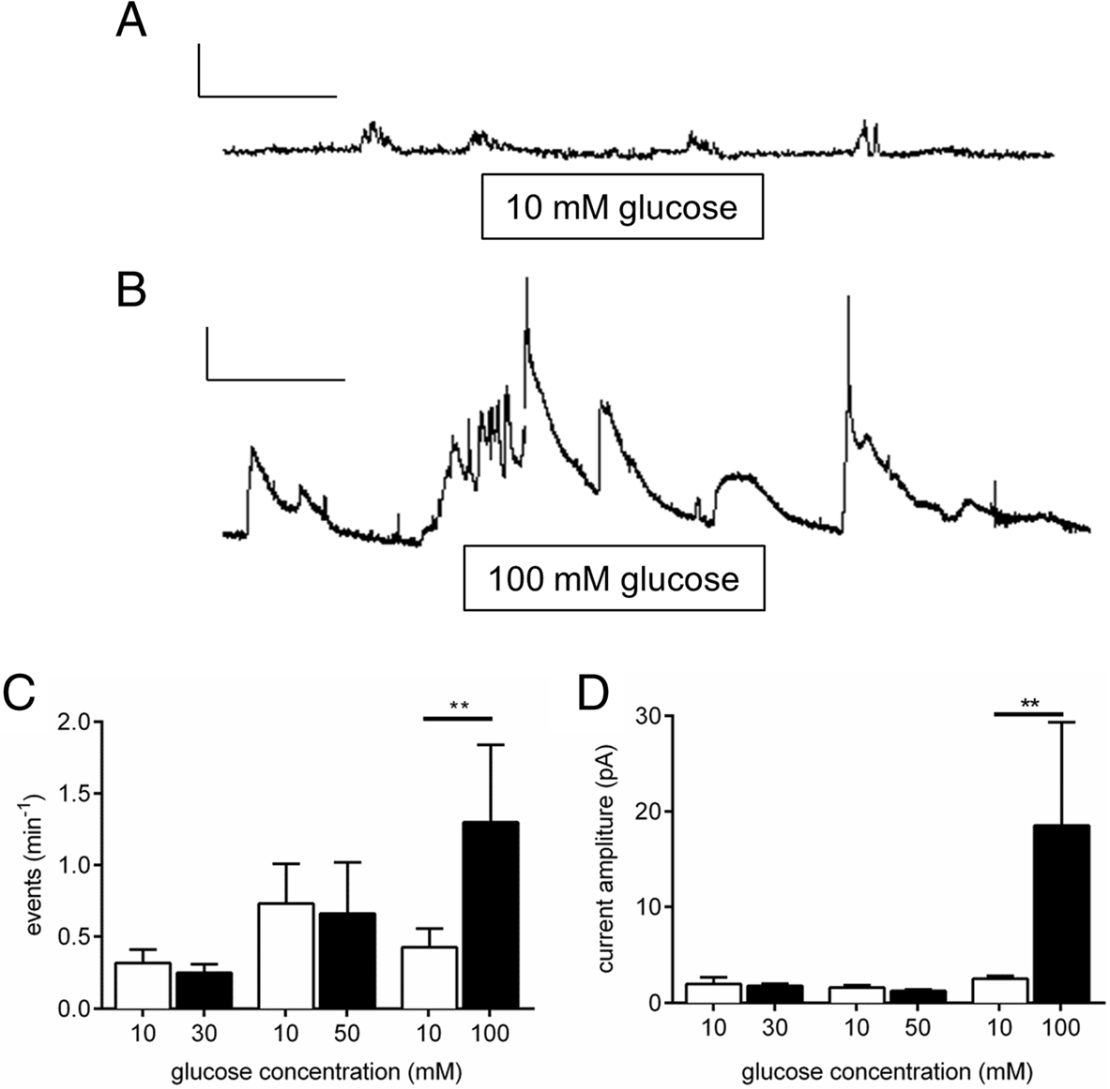
Amperometric recordings of 5-HT from intact colon. A continuous real time amperometry recording of 5-HT release in intact colon in the presence of (**a**) 10 mM glucose and (**b**) 100 mM Glucose. Scale bars represent 20 pA and 50 s. 100 mM glucose increases both (**c**) the frequency and (**d**) the size of 5-HT release events (*n* = 4, **P <0.05)

We utilised amperometry to measure 5-HT secretion in real-time in mouse colonic tissue strips to identify whether glucose can induce 5-HT release from EC cells lining the gut wall. Mice were used because they maintain regular colonic migrating motor complex (CMMC) activity and cyclical 5-HT release [22, 23] and CMMCs are less commonly recorded in vitro in other species such as guinea-pigs and rats. Under control conditions, in the presence of 10 mM glucose, we observe a frequency of 5-HT release events of ~0.5 min^−1^ (Fig. 1a). Increasing the external glucose to 100 mM causes a rapid increase in release frequency and amplitude of these release events (Fig. 1b). These changes in release frequency (Fig. 1c) and amplitude (Fig. 1d) were only significant at 100 mM glucose, with no changes seen at 20 or 50 mM.

### Acute exposure to high glucose causes Ca^2+^ entry in single EC cells

**Fig. 2:**
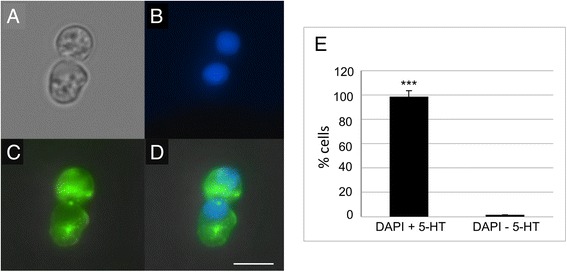
Purification of guinea pig EC cells from the colon. (**a**) bright field image of two primary EC cells in culture, (**b**) fluorescent image of the nuclear stain, DAPI, (**c**) 5-HT labelling, (**d**) merged image of 5-HT staining and DAPI. Scale bar = 4 μm. (**e**) Percentage of 5-HT positive cells in our cultures (*n* = 3 independent cell cultures)

**Fig. 3:**
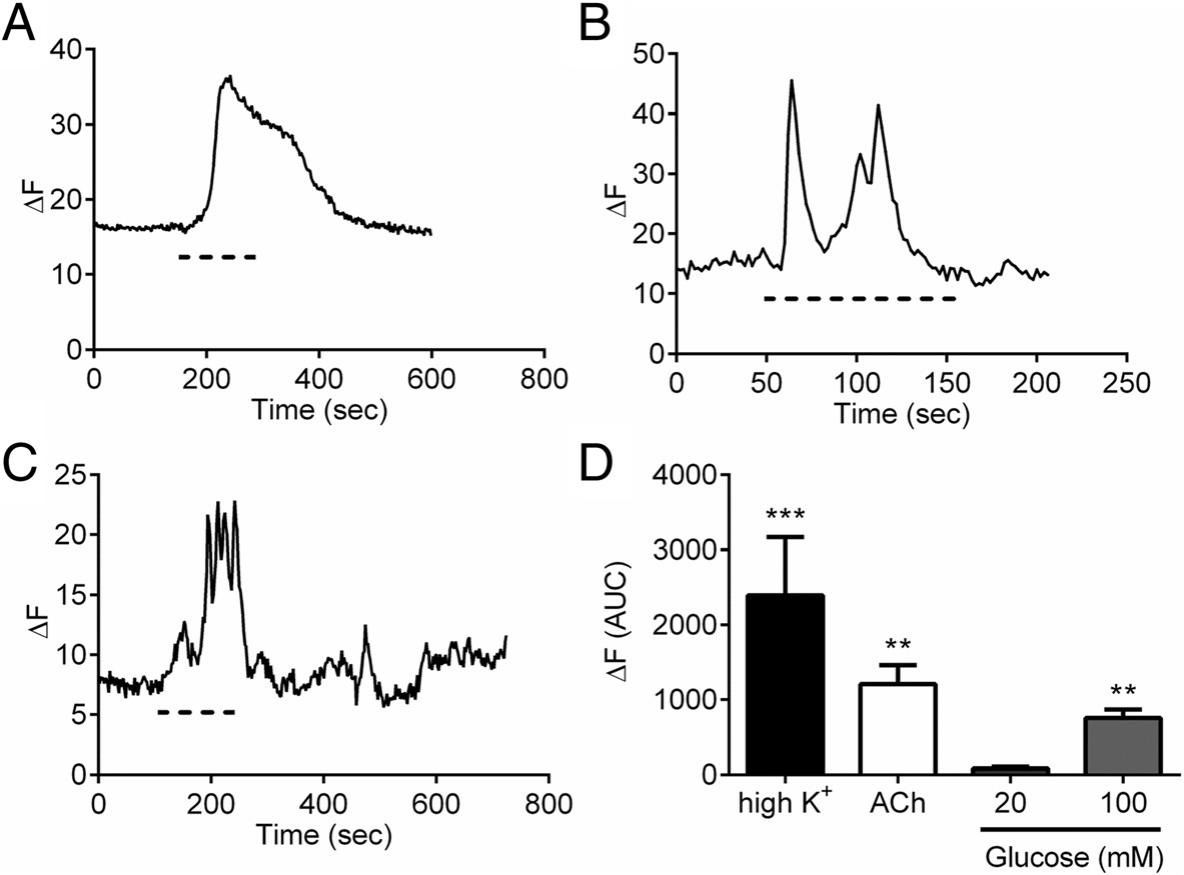
Depolarisation increases intracellular levels in EC cells. Example trace from a single EC cell representing calcium entry upon stimulation with (**a**) 100 mM K^+^, (**b**) 10 μM ACh, (**c**) 100 mM glucose as indicated with the dashed line. (**d**) Average EC cell fluorescence change in response to 100 mM K^+^ (*n* = 13 cells), 10 μM ACh (*n* = 13 cells), 100 mM glucose (*n* = 15 cells) and 20 mM glucose (*n* = 7 cells)

Intact gut tissue comprises a complex mix of different cell types including muscle, neurons, epithelial cells and endocrine cells. To examine the glucose-response in EC cells specifically, we utilised EC cells isolated from guinea-pig colon, a species and approach in which we had developed and established reliable EC cell output [17]. Using this approach we are able to isolate an almost pure population of EC cells (Fig. 2). We first performed Ca^2+^ imaging experiments on these cells to identify whether high glucose causes Ca^2+^ entry, the classical trigger of secretion in endocrine cells. As we know that high K^+^ and acetylcholine both trigger 5-HT release in these cells [17], we used these first as positive controls. We observed clear increases in intracellular Ca^2+^ upon exposure to high K^+^ (Fig. 3a) and acetylcholine (Fig. 3b). We also observed increased Ca^2+^ entry upon exposure to 100 mM glucose (Fig. 3c), but no significant Ca^2+^ increase is seen in 20 mM glucose (Fig. 3d).

### Acute exposure to high glucose augments 5-HT release from single EC cell vesicles

**Fig. 4:**
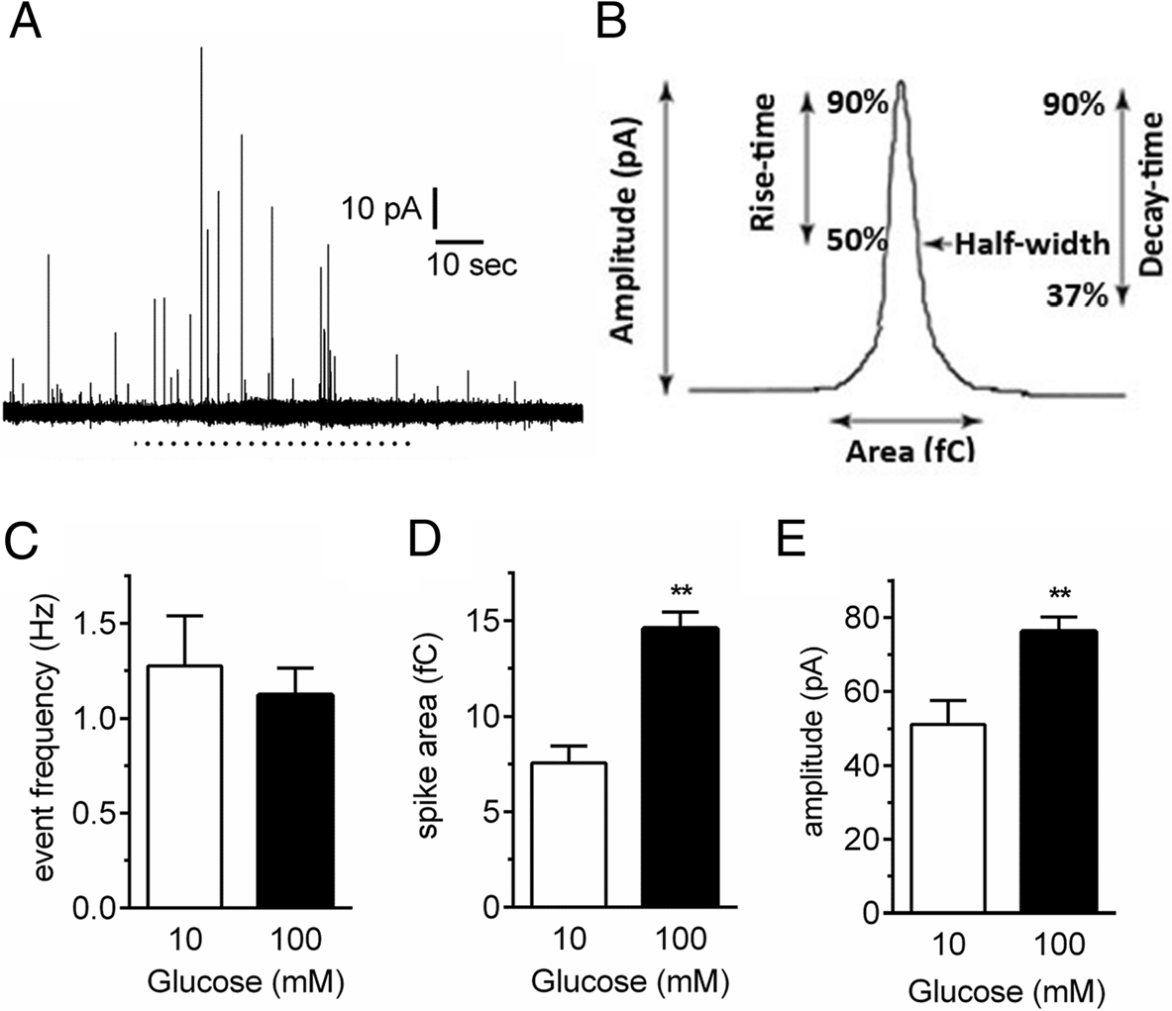
Amperometry demonstrates that high glucose increases the quantal release of 5-HT from single EC cells. (**a**) A single amperometric spike indicating the different assessable parameters including the amplitude, area, rise-time and half-width. (**b**) An example amperometric trace before and during stimulation with 100 mM glucose (dashed line). Each spike represents 5-HT release from a single vesicle. (**c**) The frequency of vesicles releasing 5-HT is unchanged by high glucose but the spike (**d**) area and (**e**) amplitude are both increased. *n* = 8 cells per group. ** *p* < 0.01

We next examined whether 5-HT secretion is increased in high glucose in our purified EC cell cultures. To do this we employed single cell amperometry, which allows us to resolve 5-HT release from individual vesicles under various conditions. Not only can we quantify the rate of vesicle exocytosis, we are also able to measure aspects of vesicle release (Fig. 4a) including the amount of 5-HT released per exocytosis event, which is measured as spike area. Given that 100 mM glucose causes 5-HT release in intact colon and Ca^2+^ entry in single EC cells, we measured whether it increases 5-HT secretion using single cell amperometry. Increasing external glucose from 10 mM to 100 mM causes an increase in 5-HT output (Fig. 4b). This is not due to an increase in the number of vesicles undergoing exocytosis (Fig. 4c), but rather to an increase in the amount of 5-HT being released per exocytosis event, as demonstrated by the significant increase in amperometric spike area (Fig. 4d) and amplitude (Fig. 4e). Thus, acute increases in glucose availability, at concentrations that would be observed post-meal, but not in circulation, trigger Ca^2+^ entry and 5-HT secretion in EC cells.

### Chronic exposure to fasting-associated levels of glucose increases Tph1 transcription and 5-HT release

**Fig. 5:**
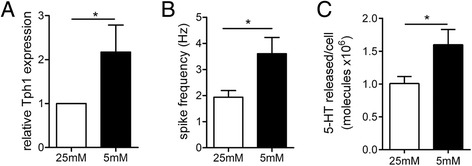
Chronic low glucose activates 5-HT release through a transcriptional pathway. (**a**) 24 h in 5 mM glucose causes and increase in Tph1 gene expression (vs 25 mM glucose, *N* = 3 preparations *n* = 9 per group. ** *p* < 0.01). (**b**) This results in an increase in 5-HT release events measured with amperometry and (**c**) more 5-HT molecules released during stimulation. *n* = 30 cells for 25 mM and 7 cells for 5 mM. *p < 0.05

It has recently been demonstrated that EC cell 5-HT is augmented during periods of fasting [6], however the identity of the nutrients associated with this change remain unknown. To identify whether reduced glucose availability may play some part in this fasting response, we incubated EC cells in concentrations that are equivalent to low (5 mM) or high blood glucose (25 mM) for 24 h to identify whether this alters 5-HT synthesis and secretion. We find that under these conditions, Tph1 gene expression is significantly higher under low glucose conditions that mimic fasting (Fig. 5a). This resulted in a significant increase in both the frequency of 5-HT release events measured using amperometry (Fig. 5b) as well as the total number of molecules of 5-HT being released from each cell (Fig. 5c). Thus, chronically reduced glucose availability causes an increase in 5-HT synthesis due to effects on Tph1 expression.

In this study we studied two aspects of enterochromaffin cell function; the nature of both the acute and chronic response to increased glucose availability. We demonstrate using intact tissue preparations and single cell approaches that acute increases in glucose, at levels found in the gut lumen rather than in plasma, trigger Ca^2+^ entry and 5-HT secretion in EC cells. Furthermore, this increased 5-HT release occurs through an increase in the amount of 5-HT released from vesicles in each exocytosis event. The effects of a more chronic exposure to high glucose, this time at levels akin to those observed in plasma post-prandially, cause a reduction in the synthesis and release of EC cell 5-HT. Thus EC cells respond in a diverse manner to different glucose concentrations over different periods of time to either increase or suppress 5-HT output.

Our data in intact colon tissue is the first *ex vivo* demonstration that EC cells are glucose-sensing cells. This is in agreement with earlier findings in BON cells that only concentrations approaching 100 mM glucose are able to elicit EC cell 5-HT release [16]. Certainly glucose infusion can alter gut motility [29, 30], and release of 5-HT in these conditions would align with the demonstrated effects of EC cell 5-HT as a modulator of gut motility [22, 23]. The fact that no response to 20 or 50 mM glucose was observed is in agreement with previous findings [16] and demonstrates that increases in plasma glucose levels are not sufficient to trigger this acute response. Rather, post-prandial levels of luminal glucose, estimated to be 200–300 mM [9], will be the only possible source of glucose capable of triggering 5-HT secretion from EC cells. Given that gut contraction is capable also of triggering EC cell 5-HT release [22, 23], we turned to a primary EC cell culture approach to determine if these effects of high glucose are through direct effects on EC cells.

We have replicated our previous work in which we demonstrated EC cell isolation in guinea-pig colon [17]. Indeed in this study we also observe an almost complete purification of EC cells. This enabled us to undertake Ca^2+^ imaging studies with the knowledge that all cells included in our analysis are EC cells. We observe a significant increase in Ca^2+^ entry upon exposure to 100 mM, but not 20 mM, glucose in line with our 5-HT secretion measurements from whole tissue. Additionally, our single cell amperometry data confirms 5-HT secretion in response to 100 mM glucose. The mechanism by which this increase occurs is somewhat surprising. Rather than observing an increase in the number of release events, we observe an increased quantal size of individual release events. Given the rapid nature of this response, this is unlikely to be an effect on vesicle loading, but rather due to an increase in either the size or open time of the exocytosis fusion pore which can be modulated by various factors [31–33].

We have previously demonstrated that the EC cell likely contains a smaller, or more rapidly closing, fusion pore that would explain the vastly lower amount released during individual release events [17]. This augmentation of quantal release is a novel mechanism of exocytosis activation that has been linked in other endocrine cells to activation of G-protein coupled receptors and second messenger signalling through PKC [34]. Whether this is the pathway activating 5-HT release in response to high glucose in EC cells remains unknown but certainly a large number of fusion pore modulators exist in endocrine cells including proteins linked to both exocytosis and endocytosis [31, 35], second messengers [36, 37] and lipids [35]. The increase in quantal release we observed could not be attributed to increased 5-HT loading into vesicles because the response was instantaneous, in contrast to the longer time base needed to load vesicles.

The final component of this work focused on trying to understand how fasting can induce an increase in gut 5-HT production [6]. This results in higher plasma 5-HT levels that are thought to be important for liberating nutrient stores such as free fatty acids and glycerol during periods of fasting [6]. To test our hypothesis that lowered plasma glucose may be a major driver of this fasting induced increased in EC cell 5-HT production, we exposed cells in culture to low or high glucose over 24 h. We observe a doubling of the expression of Tph1, the rate-limiting enzyme of 5-HT production in non-neuronal cells that is primarily expressed in EC cells and exclusively expressed within the gut in these cells. Importantly, in cells cultured in low glucose, we see functional outcomes of this transcriptional upregulation in terms of increased 5-HT release from single EC cells. Thus, low plasma glucose levels, similar to levels observed during periods of fasting, result in higher 5-HT synthesis and secretion.

## Conclusions

Thus, we have demonstrated in this study a complex interaction of EC cells with the major nutrient glucose. The pathways by which these cells respond to acute and chronic changes in glucose availability are vastly different in a number of ways. Firstly, 5-HT release is increased acutely by triggering 5-HT secretion via Ca^2+^-dependent means while the chronic glucose response augments 5-HT release in response to low glucose availability. Secondly, the effect of chronic low glucose exposure occurs through transcriptional alterations regulating 5-HT availability, while the acute response to high glucose involves the stimulus-secretion pathway. Thirdly, the concentrations of glucose underlying these two pathways reflect the acute pathway occurring due to luminal changes in glucose after a meal, while the chronic response reflects changes in plasma glucose levels over a longer time period.

These two response pathways likely serve different but important homeostatic roles. The release of 5-HT in response to an acute nutrient stimulus such as a meal will be beneficial in a number of intestinal functions of 5-HT including the modulation of gut motility, gastric emptying and movement of water and solutes across the intestinal wall. An increase in baseline plasma 5-HT levels in response to fasting and/or chronic low plasma glucose, however, will trigger the release of stored nutrients such as free fatty acids and glycerol. This represents an essential survival pathway for the organism during times of low food availability. Our findings demonstrate that EC cells release 5-HT via divergent pathways, and that the outcomes of these responses likely play important physiological functions.
